# Click chemistry facilitates direct labelling and super-resolution imaging of nucleic acids and proteins†
†Electronic supplementary information (ESI) available. See DOI: 10.1039/c4ra01027b



**DOI:** 10.1039/c4ra01027b

**Published:** 2014-12-24

**Authors:** Anika Raulf, Christoph K. Spahn, Patrick J. M. Zessin, Kieran Finan, Stefan Bernhardt, Alexander Heckel, Mike Heilemann

**Affiliations:** a Institute of Physical & Theoretical Chemistry , Goethe-University Frankfurt , Max-von-Laue-Str. 7 , 60438 Frankfurt/Main , Germany . Email: heilemann@chemie.uni-frankfurt.de; b UCL Medical School , Gower St. , London , UK; c Institute for Organic Chemistry and Chemical Biology , Goethe-University Frankfurt , Max-von-Laue-Str. 9 , 60438 Frankfurt/Main , Germany

## Abstract

We demonstrate high-density labelling of cellular DNA and RNA using click chemistry and perform confocal and super-resolution microscopy. We visualize the crescent and ring-like structure of densely packed RNA in nucleoli. We further demonstrate click chemistry with unnatural amino acids for super-resolution imaging of outer-membrane proteins of *E. coli*.

## Abstract 2

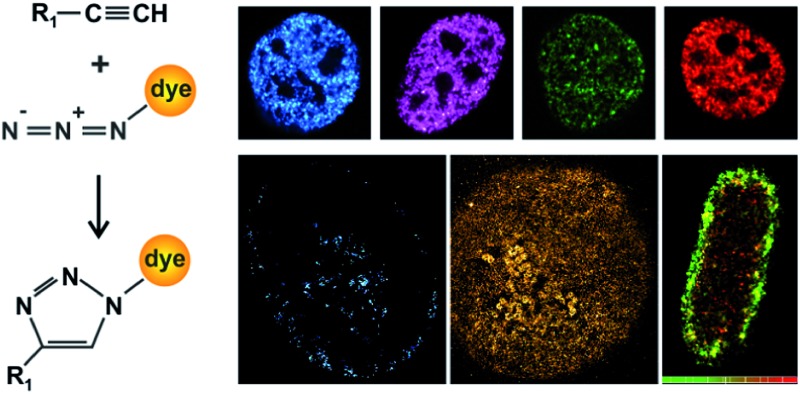
We demonstrate super-resolution imaging of proteins and nucleic acids that were densely labelled with fluorophores using the concept of “click chemistry”.

## 

Various fluorescence microscopy techniques which can bypass the spatial resolution limit have been developed in recent years. Commonly summarized as “super-resolution microscopy” techniques, they constitute a new and valuable toolbox for cell biology (for details on the different techniques, we refer to some recent reviews^1–3^). All fluorescence microscopy-based methods require suitable and specific strategies for labelling a target structure. An additional request for super-resolution microscopy is a particularly high labelling density, which is reasoned by the sampling theorem.^4^

An elegant strategy to covalently bind small synthetic fluorophores to cellular target structures with high efficiency and specificity makes use of copper(i)-catalysed Huisgen 1,3-dipolar cycloadditions,^5^ which is one example of a chemical reaction today referred to as “click chemistry”.^6,7^ This technique was initially introduced to fluorescence microscopy for labelling nucleic acids^8,9^ and phospholipids.^10^ In the recent years, the field has evolved dramatically, and new developments include alternative chemical strategies for click-like reactions, copper-free click chemistry, live cell labelling as well as dual-colour labelling with two orthogonal click reactions.^11–17^ Click chemistry has also entered the field of super-resolution microscopy, and has been applied to visualize chromosomal DNA in eukaryotes^18,19^ and *Escherichia coli* (*E. coli*)^20^ as well as proteins in the plasma membrane.^13^ A recent approach has also reported optical mapping of DNA sequences by methyltransferase-directed click chemistry and sequence readout by super-resolution microscopy.^21^

Single-molecule localization microscopy demands bright fluorophores that allow robust and well controllable photoswitching.^22^ Synthetic fluorophores exhibit a high brightness, yet require chemical functionalizations that allow labelling target structures. In addition, the chemical nano-environment (and thus also the target-specific label) of the fluorophore influences the photophysical properties^23^ or may induce quenching.^24^ So far, azide-substituted and red-emitting carbocyanines (*e.g.* Alexa Fluor 647)^18^ or custom-synthesized rhodamines^19^ were used for super-resolution imaging of DNA. Biological applications, however, require multiple targets labelled. Here, we present click chemistry labelling of cellular nucleic acids using a variety of spectrally distinct fluorophores and demonstrate confocal and single-molecule localization-based super-resolution fluorescence microscopy,^25–28^ facilitating new dye combinations for multi-colour imaging. Furthermore, we present a simple strategy to label the outer membrane proteins of *E. coli* using click chemistry in combination with an unnatural amino acid, and visualize the boundaries of bacterial cells with super-resolution imaging.

First, we explored a series of synthetic fluorophores for click chemistry labelling and aimed to find a suitable candidate that is photoswitchable if conjugated to DNA and not red-emitting. High-density labelling of DNA was achieved by addition of an alkyne-modified nucleobase (typically 5-ethynyl-2-deoxyuridine, EdU) to the growth medium, followed by natural uptake by the cells and incorporation into the chromosomal DNA during replication.^8^ After fixation and permeabilisation, the alkyne group of EdU is conjugated to an azide-substituted fluorophore through a copper-catalysed click reaction (Fig. 1A). Here, we prepared (see Materials and methods and Fig. S1 and S2†) or purchased various azide-substituted fluorophores and labelled chromosomal DNA in eukaryotic cells. We recorded fluorescence images of cells with confocal microscopy (Fig. 1B–E) and report a specific and high-density labelling of DNA in the cell nucleus for all tested rhodamine (ATTO 488, Alexa Fluor 488, Rhodamine 6G) and one carbocyanine fluorophore (Alexa Fluor 647). We explored the specificity as well as the efficiency of the target labelling by varying the reaction times with the fluorophore–azide-conjugates (Fig. S3†).

**Fig. 1:**
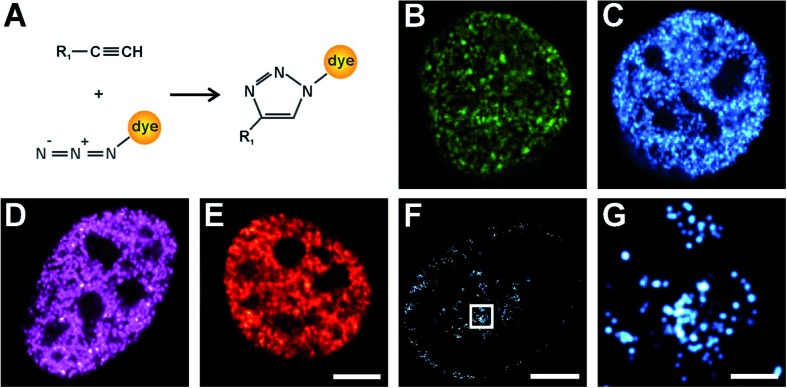
Confocal and super-resolution imaging of chromosomal DNA. (A) Alkyne-modified nucleic acids (*R*_1_ = thymidine in EdU, uracil in EU) or unnatural amino acids react in a copper-catalyzed reaction with an azide-conjugated fluorophore in a [3 + 2] cycloaddition. (B–E) Confocal images of chromosomal DNA in HeLa cells which were pulsed with EdU for 15 min, fixed and permeabilized and labelled with the fluorophore–azides ATTO 488 (B), Alexa Fluor 488 (C), Rhodamine 6G (D) or Alexa Fluor 647 (E) (scale bar 5 μm). (F) Super-resolution image of chromosomal DNA labelled with Alexa Fluor 488 in a replicating HeLa cell (scale bar 5 μm) and magnified view (G) (scale bar 0.5 μm).

So far, super-resolution imaging of DNA using bright and photoswitchable fluorophores was only demonstrated using the red-emitting carbocyanine fluorophore Alexa Fluor 647 ^18,20^ (Fig. S3†). Having fluorophores emitting at different wavelengths available for super-resolution imaging is however desirable, but often complicated by photophysical interactions with the nanoenvironment.^23,24^ Here, we demonstrate redox-induced photoswitching and single-molecule super-resolution imaging of pulse-labelled DNA labelled with the photoswitchable rhodamine fluorophore Alexa Fluor 488 (Fig. 1F and G).^29,30^ The pattern visualized in Fig. 1F is formed by newly synthesized DNA and typical for cells in the middle of DNA replication (S-phase).^31^ These results are consistent with previous work that used BrdU for pulse labelling and diffraction-limited microscopy.^32^ We determined a localization error using nearest-neighbour analysis^33^ and obtained values of 4.4 nm (Alexa Fluor 488, Fig. S4†) and of 12.3 nm (Alexa Fluor 647, Fig. S3 and S4†) (note that the higher localization error of Alexa Fluor 647 is reasoned by a lower number of photons detected in the shorter integration time of 20 ms, compared to 100 ms for Alexa Fluor 488; see Materials and methods and ref. 33). This finding suggests Alexa Fluor 488 as a suitable photoswitchable fluorophore for super-resolution imaging of nucleic acids.

Click chemistry was also reported for RNA labelling and applied to determine RNA transcription and turnover with confocal microscopy.^9^ Here, we introduce for the first time click-chemistry labelling of RNA with fluorophores compatible with single-molecule super-resolution imaging. We applied 5-ethynyluridine (EU) to pulse-label RNA of HeLa cells for 10 min and used click chemistry to label nascent RNA with Alexa Fluor 647. We visualized the nuclear RNA using confocal (Fig. 2A) and super-resolution microscopy (Fig. 2B–C). In the confocal microscopy image (Fig. 2A), high-density RNA is found within the nucleoli, where ribosomal genes are transcribed, whereas the fluorescence signal from the nucleoplasm is lower and appears rather uniform. The super-resolution image (Fig. 2B) confirms that nascent RNA transcribed by RNA polymerases II and III is distributed in distinct spots that are localized all over the nucleoplasm. The magnified image (Fig. 2C) provides additional structural information on the “hot-spots” of RNA polymerase I transcription sites visualized in the nucleoli. These structures seem to be crescent (due to optical sectioning) and rather circularly shaped around darker centres (fibrillar centres). The ribosomal RNA is surrounded by dark areas representing the condensed nucleolar chromatin. This is much expected and known from earlier work on nucleolar transcription *e.g.* visualized using electron microscopy and BrUTP immunostaining (for an in-depth discussion, we refer to ref. 34).

**Fig. 2:**
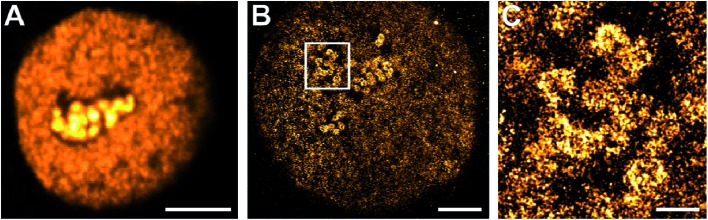
Confocal and super-resolution imaging of nuclear RNA. Living HeLa cells were exposed to EU for 10 min, then fixed with formaldehyde and permeabilized. RNA was labelled *via* click chemistry with Alexa Fluor 647 azide and imaged with confocal microscopy (A) (scale bar 5 μm) and super-resolution microscopy (B and C). (B) HeLa nucleus showing nucleoplasmic and nucleolar RNA (scale bar 5 μm). (C) The magnified inset of the cell in (B) shows crescent structures of the nucleolar RNA (scale bar 1 μm).

In comparison to other labelling techniques for nucleic acids, click chemistry with alkyne-modified nucleobases has several advantages. First, incorporation of EdU and EU leads to specific labelling only of newly synthesized DNA or RNA; nucleic acids which were already synthesized prior to EdU/EU addition will not be labelled, allowing studies of nascent replication or transcription sites similar as with BrdU or BrUTP.^34–37^ In contrast to BrdU/BrUTP, no immunostaining and additional antigen retrieval steps (*e.g.* 4 M HCL or DNase treatment) are required, which limit the application in crowded or densely packed structures such as nuclei and bacteria. Second, high labelling densities as they are required for sub-diffraction microscopy techniques can be achieved. Third, virtually any fluorophore can be conjugated to nucleic acids. This allows users to choose the optimal fluorophore for the particular application, *e.g.* photoswitchable fluorophores for single-molecule localization microscopy. DNA labelling with intercalator fluorophores compatible with super-resolution imaging has also been demonstrated.^38^ However, the photon yields and photostabilities of intercalators are lower than those of bright organic fluorophores. In some cases, the low quantum yield of unbound intercalators can also be the source of additional background signal.

An important aspect of labelling techniques applied in living organisms or cells is the biocompatibility. Both EdU and EU together were found to be non-toxic in eukaryotic cells even after exposure times of up to 20 hours^9^ and are widely used for confocal microscopy of eukaryotic cells. Here, we applied EdU/EU treatment in eukaryotic cells for 10 to 15 minutes. Furthermore, we monitored the growth rate of *E. coli* in the presence of EdU (Fig. S5†). We found no significant perturbation of the growth rate, which again suggests a rather high biocompatibility.

Click chemistry has also entered the field of protein labelling through the use of unnatural amino acids.^15–17^ This opens a novel and simple route to label the outer membrane of cells with photoswitchable fluorophores, and to visualize the boundaries of a cell with sub-diffraction resolution. We demonstrated this by visualizing the outer membrane of *E. coli*, which previously was only achieved at high contrast by phase contrast imaging.^39^ We grew *E. coli* cells in medium that contained the unnatural amino acid homopropargylglycine (HPG). During cell growth, HPG is incorporated into newly synthesized proteins, replacing methionine, which facilitates the subsequent labelling of all newly synthesized proteins with an azide–fluorophore^17^ (Fig. 3A). In order to specifically label the proteins on the outer membrane, cell integrity has to be maintained. We therefore chose to apply a fixation protocol which does not disrupt the outer membrane, so that staining of cytosolic proteins is prevented. Hence, we labelled newly synthesized proteins on the outer membrane which expose an alkyne group. Tagged with a suitable fluorophore for single-molecule super-resolution microscopy, this simple approach allows visualizing of the bacterial outer membrane with high resolution, thus providing a sharp image of the contour of a bacterial cell (Fig. 3B). The resulting image allows determination of the bacterial length and width. 3D-imaging would allow the visualization of the cylindrical shape, which would be a viable reference structure.^40^ In addition, this approach is in principle live-cell compatible; incorporated azide-containing amino acids could be labelled in copper-free click-reactions.^11,12^

**Fig. 3:**
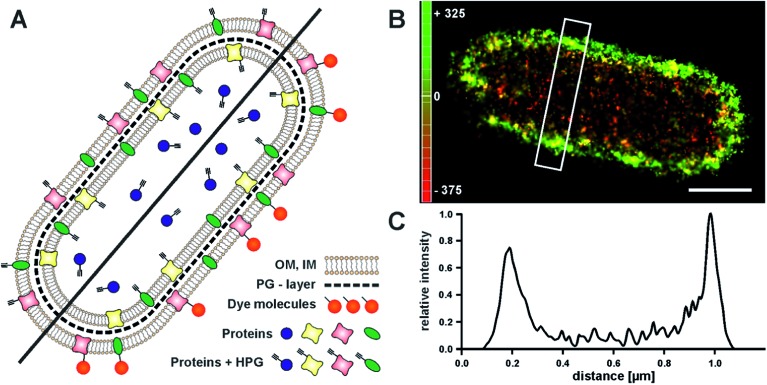
Super-resolution imaging of the *E. coli* outer-membrane proteins. (A) The methionine-analogue homopropargylglycine (HPG) is incorporated into newly synthesized proteins. Click reaction with an azide exclusively labels HPG residues outside the cell, resulting in an outer-membrane (OM) stain. Cytosolic or inner-membrane (IM) proteins are separated by the outer membrane and the peptidoglycan layer (PG), and are not labeled. (B) 3D *d*STORM image of a HPG-treated *E. coli* cell, labeled with Alexa Fluor 647. The *z*-position is color-coded and indicates the cylindrical shape of the bacterial cell. (C) Cell diameters can be determined from the intensity profile of a cross-section (white rectangle in (B)) (0.8 μm for the cell in (B)) (scale bar 0.5 μm).

In summary, we have demonstrated click chemistry as versatile tool for super-resolution fluorescence microscopy of cellular structures. Click chemistry on the one hand can provide the high labelling densities typically required for super-resolution microscopy. On the other hand, it is a flexible strategy to label nucleic acids with virtually any synthetic fluorophore, which allows choosing the best-suited one for a specific super-resolution technology. Furthermore, this approach can be combined with pulse labelling and thus visualize particular states of the cell cycle. Finally, a whole new area of applications opened with the introduction of click chemistry into protein labelling through unnatural amino acids. In the future, click chemistry will certainly develop into a widespread and generally used toolbox for cell and tissue labelling.
