# Supplementary material for: Click chemistry facilitates direct labelling and super-resolution imaging of nucleic acids and proteins
Source: RSC Adv. 2014 Dec 24;4(57):30462–6. doi: 10.1039/c4ra01027b (PMC4285124; doi:10.1039/c4ra01027b)
Supplement: Supplementary file 1 [file RA-004-C4RA01027B-s001.pdf]

Electronic Supplementary Material (ESI) for RSC Advances.
This journal is © The Royal Society of Chemistry 2014

Click chemistry facilitates direct labelling and super-resolution imaging of nucleic acids and proteins

*Anika Raulf,[a] Christoph K. Spahn,[a] Patrick J. M. Zessin,[a] Kieran Finan,[b] Stefan Bernhardt,[c] Alexander Heckel[c] and Mike Heilemann[a, *]*

[a] Institute of Physical & Theoretical Chemistry, Goethe-University Frankfurt, Max-von-Laue-Str. 7, 60438 Frankfurt/Main, Germany

[b] UCL Medical School, Gower St., London, United Kingdom

[c] Institute for Organic Chemistry and Chemical Biology, Goethe-University Frankfurt, Max-von-Laue-Str. 9, 60438 Frankfurt/Main, Germany

[*] Corresponding author: heilemann@chemie.uni-frankfurt.de

SUPPLEMENTAL INFORMATION

MATERIALS AND METHODS

Preparation of fluorophore-azide conjugates. The fluorophores Alexa Fluor 488 and Rhodamine 6G (Life Technologies) were purchased as N-succinimidyl (NHS) esters. The NHS-fluorophores were coupled to the carboxyl-reactive building block 3-azido-1-propanamine (Sigma, Germany) in anhydrous DMF (Sigma, Germany) and triethylamine (Sigma, Germany) (1:4 ratio) at 30 °C over night. The solvent was evaporated, and the reaction mixture re-diluted in 9:1 water:acetonitril (Sigma, Germany) for HPLC purification. Reversed phase HPLC (PU2080, Jasco, Germany) was performed using an analytical C18-column (Nucleosil 100-5 C18, Macherey-Nagel, Germany) and a gradient of acetonitril and water as mobile phase. Elution was done with an increasing acetonitril gradient (see Fig. S1 and S2 for characterization of dye-azides). The purified fluorophore azides were evaporated, re-dissolved in DMSO (Sigma) and stored at -20 °C. The fluorophore-azides of ATTO 488 (baseclick, Germany) and Alexa Fluor 647 (Life Technologies) were purchased.

Cell culture. HeLa cells were seeded in 8-well chamber slides (Sarstedt, Germany) and grown for 24h in DMEM high glucose complemented with 1% GlutaMAX (Gibco) and 10 % fetal bovine serum (Biochrom AG, Germany) at 37°C, 5 % CO_2.

Sample preparation. For DNA labelling, EdU (5-ethynyl-2-deoxyuridine; Invitrogen, Oregon, USA) was added to HeLa cells to a final concentration of 10 µM. After 15 min cells were fixed with 4 % formaldehyde solution (Sigma, Germany) in phosphate buffered saline (PBS; Sigma) for 10 min. Cell membranes were permeabilised with 0.5 % Triton X-100 (Sigma) for 30 min, afterwards click reaction was performed to label the alkine group of EdU with the indicated fluorophore azides using 5 µM azide in 100 mM Tris pH 8.5 (Sigma), 1 mM $CuSO_4$ (Sigma) and 100 mM L-ascorbic acid (Sigma).

For RNA labelling, Hela cells were incubated with 1.7 mM EU (5-ethynyluridine; Invitrogen, Oregon, USA) in growth medium for 10 min at 37°C, 5 % CO_2. Fixation, permeablisation and click chemistry were performed as described for DNA labelling. Afterwards cells were incubated with 0,01 % Triton X-100, 1 M NaCl (Sigma) in RNA*later* RNA stabilization reagent (Qiagen) overnight to remove free fluorophores. Buffers were prepared in H_2O or PBS treated with 0.1 % diethyl pyrocarbonate (Sigma, Germany) for 1h at 37 °C and autoclaved, further 1 µl/ml RNaseOUT™ recombinant RNase inhibitor (Life technologies) was added to the click reaction. The 8-well chamber slides were placed on ice and buffers were pre-chilled for all steps

except for click reaction which took place at room temperature. Samples were stored in RNA*later* RNA stabilization reagent at 4°C.

Proteins on the outer membrane of the *E. coli* strain KF26 (1) were labelled by growing bacterial cells in the presence of 250 µM homopropargylglycine (HPG) (Life Technologies) at 32°C and 200 rpm. When cells reached the logarithmic phase (OD_{600} ~ 0.3), they were transferred to a minimal growth medium (M9) to deplete the methionine pool. After 30 min growth in M9, HPG was added for 1 h. Fixation, click reaction with Alexa Fluor 647 azide (Life Technologies), and immobilization on 8-well chamber slides (Sarstedt) was performed as described elsewhere (2).

Confocal microscopy. Confocal images were recorded with a commercial confocal microscope (LSM710, Zeiss, Germany) with appropriate laser excitation and filter settings. Samples were imaged in PBS.

Super-resolution microscopy. Super-resolution microscopy was performed either on a commercial system (N-STORM, Nikon, Japan), or on a custom-built microscope as described earlier (3). Briefly, an inverted microscope (Olympus IX71) was equipped with a 100x oil objective (PLAPO 100x TIRFM, NA ≥ 1.45, Olympus) suitable for total internal reflection fluorescence (TIRF) microscopy. Appropriate filter sets for excitation and emission were used, and the fluorescence light projected onto an EMCCD camera (Ixon3, Andor, Ireland). Typically, between 15000 and 20000 frames were recorded at integration times of 100 ms (20 ms for Fig. S3). Samples labelled with Alexa Fluor 488 were irradiated at 488 nm (Sapphire 488 LP, Coherent) and samples labelled with Alexa Fluor 647 at 643 nm (iBeam smart, Toptica Photonics).

Super-resolution imaging of Alexa Fluor 647-labeled DNA and RNA was performed in PBS and 100 mM beta-mercaptoethylamine (MEA) (Sigma, Germany) at pH 8.0. Alexa Fluor 488-labelled DNA was imaged in 100 mM MEA at pH 7.5 in PBS. *E. coli* cells were imaged in Tris-buffer (pH7.5) containing 100 mM MEA and an enzymatic oxygen scavenger system as published elsewhere (4).

Super-resolution data was analyzed with rapidSTORM (5) or NIS-Elements (version 4.00.01., Nikon, Japan).

SUPPLEMENTAL FIGURES

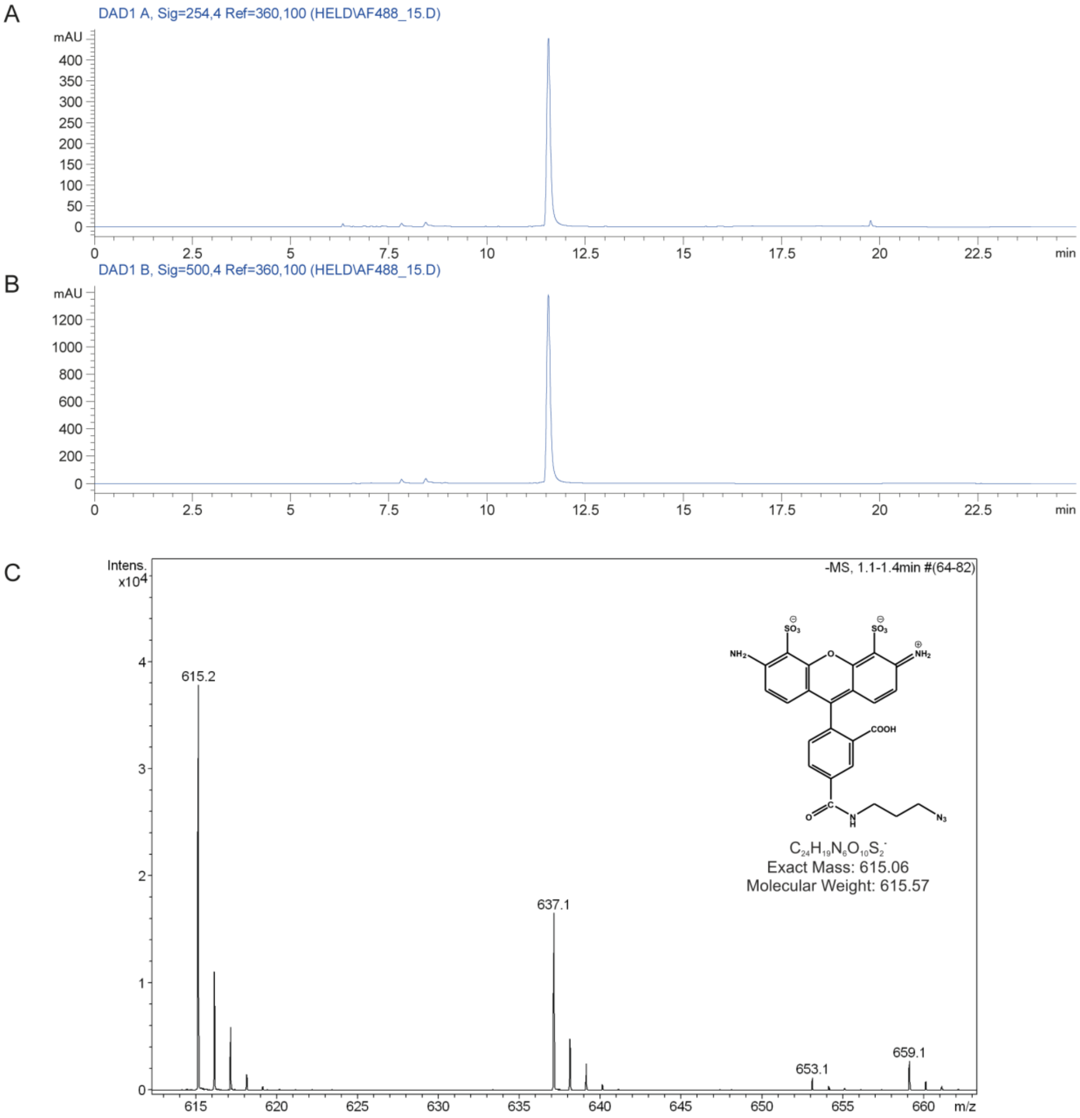

Fig. S1 Characterization of the Alexa Fluor 488 azide. (A) HPLC trace (detection at 254 nm, MultoKrom 100-5 C18, 250 mm x 4.6 mm, solvent A: 0.01% TFA in H_2O, solvent B: MeCN, gradient: 0%B → 40%B in 12 min., flow: 1 mL/min); (B) HPLC trace (conditions same as under A but detection at 500 nm); (C) result of ESI-TOF-MS analysis and chemical structure.

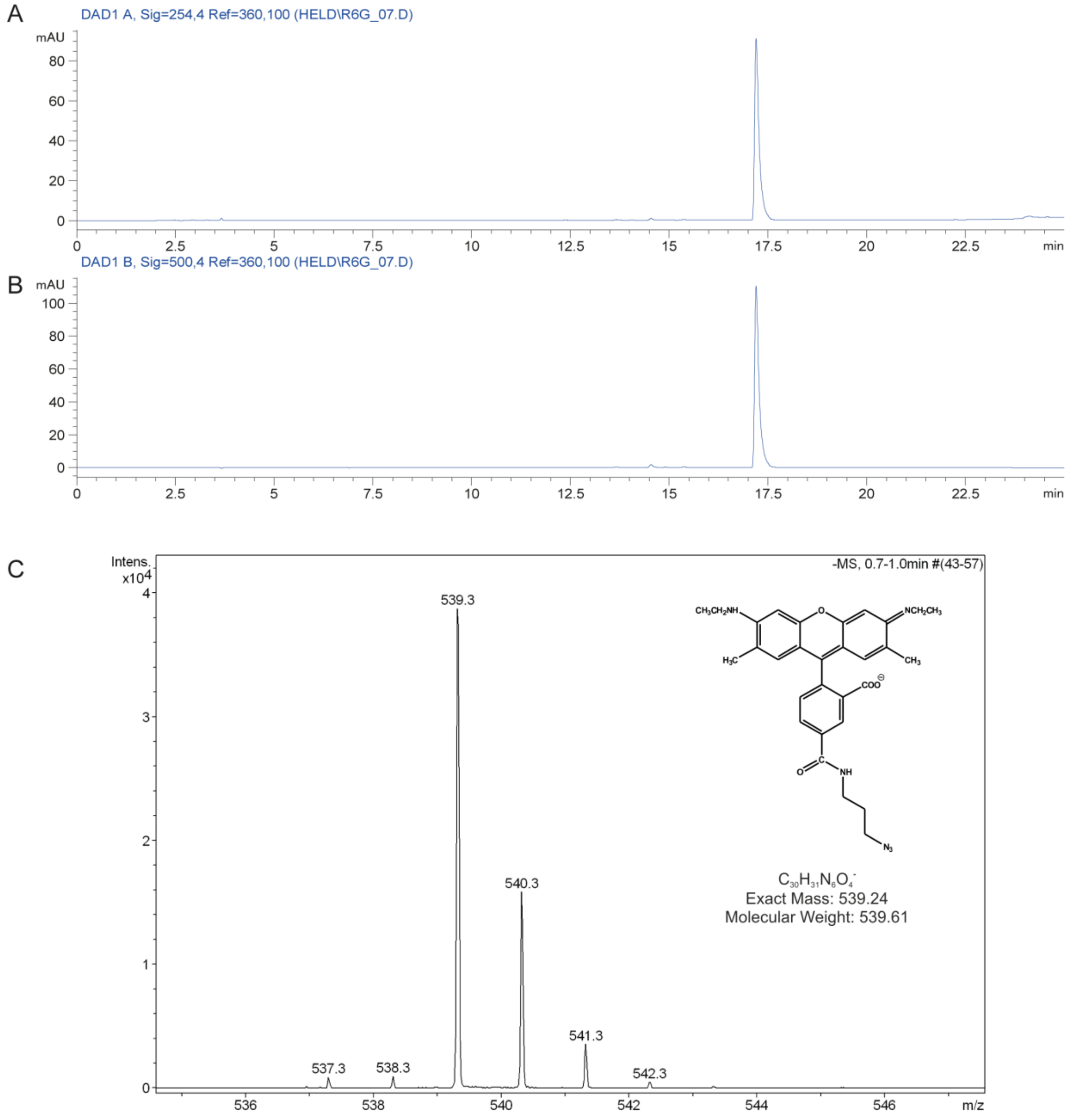

Fig. S2 Characterization of the Rhodamine 6G azide. (A) HPLC trace (detection at 254 nm, MultoKrom 100-5 C18, 250 mm x 4.6 mm, solvent A: 0.01% TFA in H_2O, solvent B: MeCN, gradient: 20%B → 60%B in 19 min., flow: 1 mL/min); (B) HPLC trace (conditions same as under A but detection at 500 nm); (C) result of ESI-TOF-MS analysis and chemical structure.

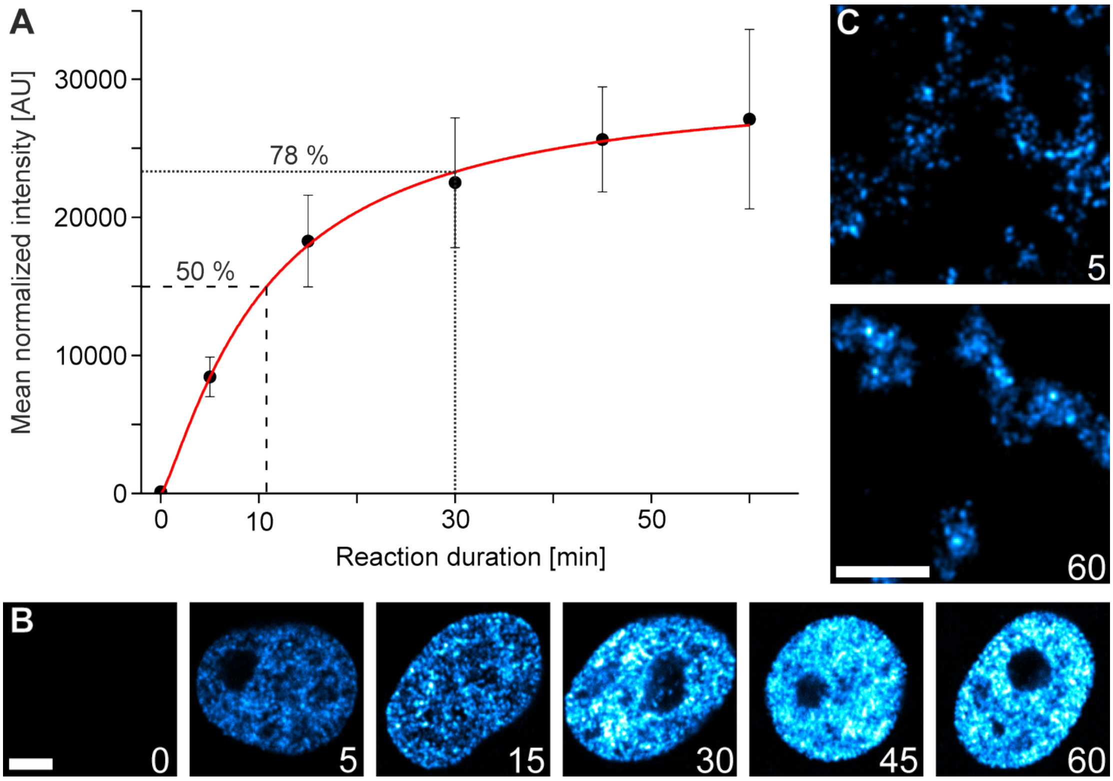

Fig. S3 Labelling efficiency of the click-reaction over time. HeLa cells were grown and pulse-labelled with EdU for 15 minutes and conjugated to Alexa Fluor 647. The reaction time of the click reaction was varied between 5 and 60 minutes. (A) The average intensity per nucleus (N = 30-50 cells) was measured with Fiji for each reaction time. The data was fitted with a Hill function to calculate the reaction constant of the intra nuclear click reaction (k = 10.73 min) and the total efficiency after 30 minutes of buffer incubation (78 %). (B) Exemplary confocal images of for different reaction times (scale bar 5 µm). (C) Super-resolution images of chromosomal DNA (mid-phase replication) acquired after 5 and 60 minutes reaction time, respectively (scale bar 500 nm, exposure time 20 ms). A lower labelling density is visible for a reaction time of 5 minutes.

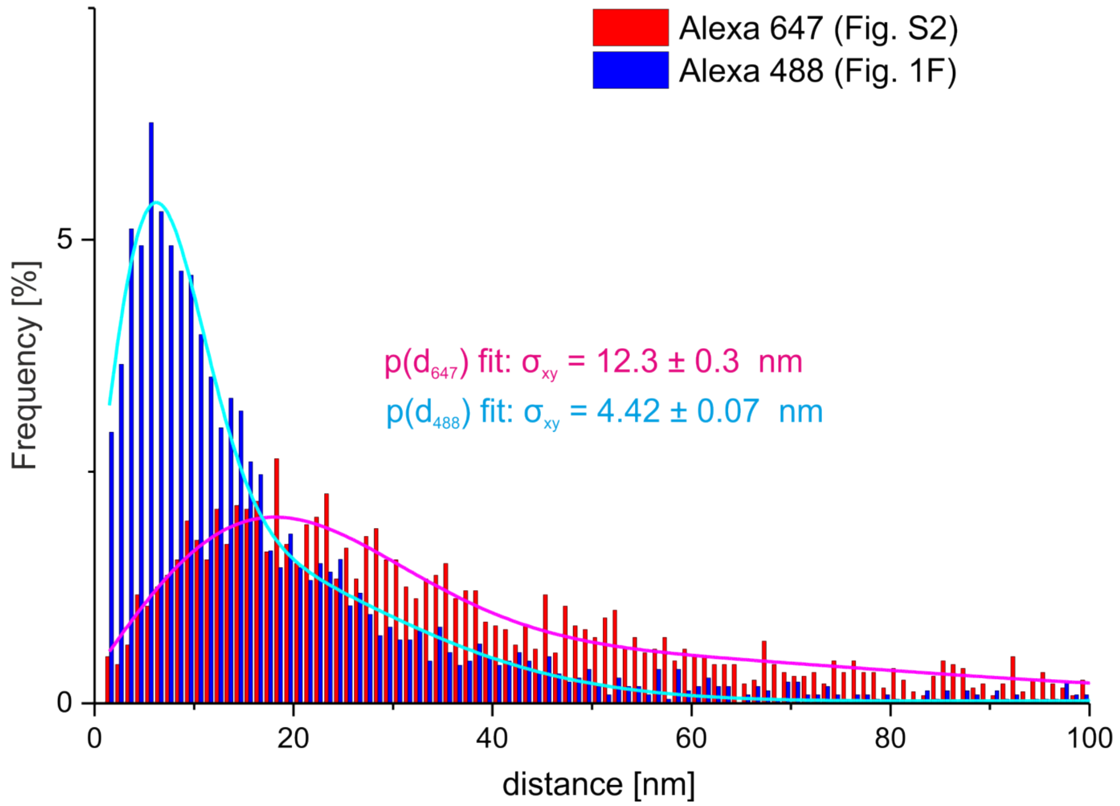

Fig. S4 Localization precision of super-resolution images shown in Fig. 1F (labelled with Alexa Fluor 488) and S2 (labelled with Alexa Fluor 647). The localization error σ_{xy} was calculated with a nearest-neighbor approach (6). For Alexa Fluor 647 (Fig. S3) and for Alexa Fluor 488 (Fig 1F), a localization error of 12.3 nm and 4.42 nm was determined, respectively. The higher localization error determined for Alexa Fluor 647 is reasoned by a shorter integration time per frame (20 ms, over 100 ms for Alexa Fluor 488).

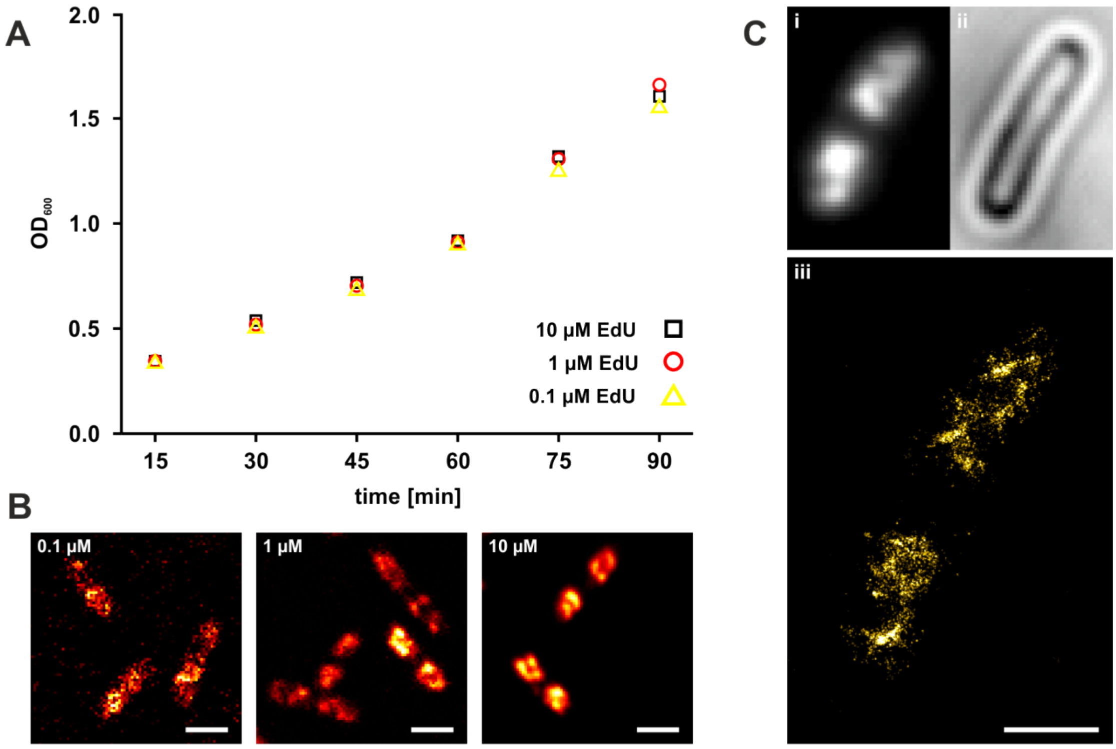

Fig. S5 Influence of various concentrations of EdU on bacterial growth. Cells were grown at 32°C in LB. (A) Doubling times did not change significantly over more than two mass doublings (35.56, 34.26 and 35.19 min for 10, 1 and 0.1 µM EdU, respectively). (B) Confocal microscopy images of DNA click-labelled with Alexa Fluor 647 show increasing intensity as expected (brightness and contrast was increased for 0.1 µM, for better visualization). (C) dSTORM image of Alexa Fluor 647 labeled *E. coli*, incubated with 10 µM EdU. The highly resolved image (iii) reveals much more details as seen in the diffraction limited widefield image (i) or brightfield image (ii) of the *E. coli* cell (ii) (scale bars 1 µm).

REFERENCES

1. Endesfelder U, Finan K, Holden SJ, Cook PR, Kapanidis AN, Heilemann M. Multiscale spatial organization of RNA polymerase in Escherichia coli. Biophys J. 2013;105(1):172-81. Epub 2013/07/05.
2. Spahn C, Endesfelder U, Heilemann M. Super-resolution imaging of Escherichia coli nucleoids reveals highly structured and asymmetric segregation during fast growth. J Struct Biol. 2014. Epub 2014/01/30.
3. Dietz M, Fricke F, Krüger C, Haase D, Niemann H, Heilemann M. Receptor–Ligand Interactions: Binding Affinities Studied by Single-Molecule and Super-Resolution Microscopy on Intact Cells. Chemphyschem. 2013:DOI: 10.1002/cphc.201300755.

4. Dempsey GT, Vaughan JC, Chen KH, Bates M, Zhuang X. Evaluation of fluorophores for optimal performance in localization-based super-resolution imaging. Nat Methods. 2011;8(12):1027-36. Epub 2011/11/08.
5. Wolter S, Schuttpelz M, Tscherepanow M, S VDL, Heilemann M, Sauer M. Real-time computation of subdiffraction-resolution fluorescence images. J Microsc. 2010;237(1):12-22. Epub 2010/01/09.
6. Endesfelder U, Malkusch S, Fricke F, Heilemann M. A simple method to estimate the average localization precision of a single-molecule localization microscopy experiment. Histochemistry and cell biology. 2014. Epub 2014/02/14.
